# Off-pump sutureless repair for ischemic left ventricular free wall rupture: a systematic review

**DOI:** 10.1186/s13019-017-0603-7

**Published:** 2017-05-19

**Authors:** Yoshio Misawa

**Affiliations:** Division of Cardiovascular Surgery, Jichi Medical University, 3311-1 Yakushiji, Shimotsuke, Tochigi 329-0498 Japan

**Keywords:** Ischemic left ventricular rupture, Sutureless repair, Left ventricular pseudoaneurysm, TachoComb®, TachoSil®

## Abstract

**Background:**

Clinical results of ischemic left ventricular free-wall rupture show high mortality rates.

**Methods:**

We reviewed studies published after 1993 on PubMed.

**Results:**

A sutureless technique using fibrin glue sheets or patches with/without fibrin glue might contribute to improved clinical results. However, some technique limitations remain for blowout-type ruptures, and the possibility of a pseudoaneurysm formation at the repair site after surgery should be considered.

**Conclusions:**

The sutureless technique can be a promising strategy for the treatment of ischemic rupture, but serial echocardiographic studies should be mandatory for diagnosing a left ventricular pseudoaneurysm formation thereafter.

## Background

Cardiac rupture after acute myocardial infarction, which includes ventricular free wall rupture, septal rupture, and papillary muscle rupture, could result in lethal complications. Post-infarction ventricular septal defects develop in approximately 1%-3% of patients prior to the advent of thrombolytic therapy and percutaneous coronary artery interventions. Thereafter, the frequency is substantially reduced to less than 0.5% of patients with acute myocardial infarction [1].

Mitral valve regurgitation caused by rupture of a papillary muscle presents in 1%-3% of patients. The posteromedial papillary muscle ruptures in about 75% of these patients and the anterolateral muscle in about 25%, although complications are less common given the widespread use of thrombolytic and percutaneous intervention therapy [1]. After total papillary muscle rupture, only about 25% of patients treated non-surgically survive for more than 24 h. Surgical treatment of patients with total rupture results in poor outcomes and high mortality. Schroeter et al. recently showed a 30-day mortality rate of 39.3% among 28 patients with papillary muscle rupture [2].

These life-threatening complications mainly occur within 7 days after myocardial infarction. Free-wall rupture of the left ventricle is another mechanical complication that can occur after acute myocardial infarction. However, its clinical outcomes are poorly studied because of the rarity of the condition. Here, the author reviews surgical results of the ischemic free-wall rupture of the left ventricle, particularly after sutureless repair.

As conventional procedures, direct suture closures of rupture sites are employed under cardiopulmonary bypass. Because of the rapid deterioration of these patients with free-wall rupture, the mortality rate within the first week is very high if left untreated. Patients can extend their 5-year survival rates to 65% with conventional surgical corrections including infarctectomy with patch reconstruction, direct closure with or without patch covering, and endoventricular patch repair under cardiopulmonary bypass [3]. However, early postoperative mortality remains high. Iemura et al. reported on operative results from 17 patients including 13 with oozing-type rupture, concluding that the overall surgical mortality rate was 11.8% [4]. McMullan et al. treated 18 patients including 14 with blowout rupture, showing that 11 patients (61%) died after surgery [5]. They also reported four cases associated with re-rupture 1 to 12 h after infarctectomy and direct suture closure were reported, suggesting the limitations of suture repair surgery.

Reardon and colleagues reported that free wall rupture was a complication after myocardial infarction in 4% to 24% of patients and that it was the second most common cause of death (pump failure being the most common cause), accounting for 12% to 21% of deaths after myocardial infarctions [6]. Repair of ischemic free wall rupture in Japan has recently been associated with a high mortality rate of 33% to 38% [7, 8]. Unfortunately, the details of these procedures are unknown.

## Methods

We reviewed studies written in English published after 1993 on PubMed, including studies written in non-English with English abstracts as of the end of 2015. Studies were searched for using following key words: ischemic left ventricular rupture, sutureless repair, left ventricular pseudoaneurysm, left ventricular repair, and sutureless repair.

## Results

Thirteen articles matched the key words, and they were reviewed. Patients’ profiles, operative procedures, and outcomes are summarized in Tables 1 and 2. Oozing type ruptures were mainly reported. Thirty-five cases including 33 oozing and 2 blowout ruptures were analyzed. Preoperative pericardial drainage was performed in some cases. The anterior wall lesions were recognized in 11 cases, the lateral wall in 13 cases, and the inferior or posterior in 11 cases. Perioperative intra-aortic balloon pumping was also employed in several cases. Twenty-three cases were repaired without cardiopulmonary bypass. Patch materials have been changed from Teflon and autologous pericardium to TachoComb^®^ (CSL Behring, Tokyo, Japan) and TachoSil^®^ (Baxter Healthcare Corporation, Dearfield, IL, USA).

**Table 1:** Patients’ profiles-1

article	First Author	Journal#1	Year	Cases	Rupture type	Per. Drain.#2	rupture site#3	IABP#4
1	Padró	ATS	1993	13	oozing	Yes in 8	3 Ant. 3 Lat. 7 Inf. or Post.	N.A.
2	Iha	ATCS	2001	1	oozing	No	Ant.	N.A.
3	Alamanni	JTCS	2001	4	oozing	No	4 Lat.	N.A.
4	Lachapelle	ATS	2002	6	3 bleeding 2 sealed 1 oozing	Yes in some	3 Lat. 2 An.t. 1 Inf.	Yes in 2
5	Fukushima	ICTS	2003	1	oozing	Yes	Antero-lat.	No
6	Nishizaki	JJTCS	2004	1	blowout	No cardiogenic shock	Ant.	Yes
7	Matsushita	KG	2004	1	oozing	No	Ant.	No
8	Kimura	JJTCS	2005	1	blowout	No cardiogenic shock	Antero-lat.	No
9	Muto	ATS	2005	1	oozing	No cardiogenic shock	Lat.	No
10	Aoyagi	JCS	2014	3	oozing	Yes in 2	Post. Lat. Inf.	Yes
11	Isoda	ATCS	2014	1	oozing	Yes	Ant.	Yes
12	Sasaki	GTCS	2014	1	oozing	Yes	Lat.	No
13	Kurumisawa	KG	2015	1	oozing	No	Inf.	No

Abbreviations

#1 *ATS* Ann Thorac Surg, *ATCS* Ann Thrac Cardiovasc Surg, *JTCS* J Thorac Cardiovasc Surg, *ICTS* Interact Cardiovasc Thorac Surg, *JJTCS* Jpn J Thorac Cardiovasc Surg, *KG* Kyobu Geka, *JCS* J Card Surg, *GTCS* Gen Thorac Cardiovasc Surg

#2 *Per. Drain*. pericardial drainage

#3 *Ant.* anterior wall of the left ventricle, *Lat.* lateral wall of the left ventricle, *Inf.* inferior wall of the left ventricle, *Post.* posterior wall of the left ventricle, *Antero-lat.* antero-lateral wall of the left ventricle

#4 *IABP* perioperative intra-aortic balloon pumping, *N.A.* not available

**Table 2:** Patients’ profiles-2

article	CPB#1	Patch material	Additional material	PA formation#2	Follow-up	Outcomes
1	No in 12	Teflon	Histoacryl	No	up to 5 years	surviving
2	No	Autologous pericardium	GRF glue	Yes 24 months	28 months	surviving after reop#3
3	Yes in 3	Glubran/Autologous pericardium	BioGluel	No	3-22 months	surviving
4	Yes in 4	Teflon	Histoacryl	No	2 months -7.5 years	5 surviving
5	No	TachoComb	none	Yes 1 year	1 year	surviving after reop
6	No	TachoComb	none	No.	18 days	surviving
7	PCPS	none	Fibrin glue/Surgicel	No.	6 years	surviving
8	PCPS	TachoComb	none	Yes 7 days	50 days	surviving after reop
9	No	TachoComb	none	No.	15 months	surviving
10	No	TachoComb	Gelfoam	Yes 4 months	16 months	1 surviving after reop, 1 died of re-reupture 10 days after surgery
11	No	TachoComb	GRF Glue	No	12 months	surviving
12	No	TachoComb	none	Yes, 8 days	25 days	surviving after reop
13	No	TachoSil	Surgicel	No	20 month	surviving

Abbreviations

#1 *CPB* cardiopulmonary bypass, *PCPS* percutaneous cardio-pulmonary support, #2 *PA formation* Left ventricular pseudoaneurysm formation, #3 *reop*: reoperation

Teflon: Boston Scientific, Meadox Medical Inc, Oakland, NJ, USA, Glubran; GEM Inc, Viareggio, Italy, TachoComb :CSL Behring, Tokyo, Japan, TchoSil: Baxter Healthcare Corporation, Dearfield, IL, USA

Histoacryl: B. Braun Medical AG, Melsungen, Germany, GRF glue:, Nippon BXI Inc., Tokyo, Japan

BioGlue; CryoLife International, Inc, Kennesaw, GA, USA, Surgicel: Ethicon; Somerville, NJ, USA

Padró et al. reported 13 successful cases treated by sutureless repair [9]. They applied a polytetrafluoroethylene patch over the infarcted area that was attached to the heart surface with surgical glue. All patients survived the surgery and were discharged from the hospital at a mean of 15 days after surgery. Follow-up extending up to 5 years in total showed 100% survival. Lachapelle et al. treated six unstable hemodynamic cases with the free-wall rupture, resulting in five survivors between 2 months and 7.5 years [10]. Aoyagi et al. also reported three successful cases treated with a fibrin glue sheet and an absorbable gelatin sponge [11]. Other investigators described cases treated with an off-pump sutureless procedure using a fibrin glue sheet with or without glue [12–18].

Following the sutureless procedures, several studies have reported on pseudoaneurysm formation of the left ventricle. Kimura et al. described a case with such a complication, who developed a left ventricular pseudoaneurysm 7 days post-surgery [19]. The pseudoaneurysm was successfully repaired under a cardiopulmonary bypass. The authors warned that sutureless repair should be avoided when treating a blowout-type rupture.

Following the sutureless procedures, several studies have reported on pseudoaneurym formation of left ventricle [20–22]. The reported incidence of pseudoaneurym was 14.3% (5/35), and these 5 cases were reported to occur between 7 days and 2 years. Pseudoaneurysm formation of the left ventricle after sutureless repair is also shown in Table 2. These pseudoaneurysms were diagnosed between 7 days and 24 months after the repair surgery, and all patients were successfully repaired with patches such as the Dor procedure [23]. Additional complications included the development of mitral papillary muscle rupture or ventricular septal perforation after sutureless repair by two patients [17, 18]. Another patient suffered from re-rupture of the repaired left ventricle [11].

## Discussion

Nasir et al. reviewed articles that analyzed the outcomes related to conservative and surgical approaches and the effects of cardiopulmonary bypass under systemic heparinization [24]. They concluded that patients with a milder form of rupture could be managed conservatively, but that those patients were at risk of developing a large defect. They also mentioned that the sutureless procedure involving the patch and glue technique enables cardiopulmonary bypass to be avoided and improves short and midterm survival rates.

Sutureless procedures are somewhat different among investigators, and materials applied to myocardial lesions have been changed. Typically, the heart is accessed through a standard sternotomy and pericardiotomy, and several layers of fibrin glue sheets or patches with or without fibrin glue are applied to the rupture site including the infarcted area. The oozing surface is then compressed for several minutes to confirm complete hemostasis. Many surgeons perform this procedure without the need for cardiopulmonary bypass.

Ischemic rupture can occur at any portion of the left ventricle (Table 1). Pericardial drainage is not always performed in the case of deteriorated hemodynamic conditions, which require immediate pericardial exploration. Perioperatively, intra-aortic balloon pumping is sometimes used to reduce the afterload of the left ventricle. Cardiopulmonary bypass tends to be avoided because systemic heparinization may disturb the hemostatic procedures of sutureless repair.

Patch materials vary from an expanded polytetrafluoroethylene membrane such as Teflon to an equine collagen sponge coated with a solid component of fibrin glue such as TachoComb. TachoComb should be applied to myocardium covering infarcted myocardium and healthy myocardium surrounding to reduce the shear stress of the infarcted myocardium. Many surgeons repeated this procedure several times and they applied additional glue drops to the area of myocardial rupture. The sutureless technique, which employs surgical glue sheets such as TachoComb, has become widespread.

After sutureless repair of ischemic cardiac rupture, a pseudoaneurysm of the left ventricle can be encountered. The pseudoaneurysm can spontaneously occur after myocardial infarction. Surgical mortality and long-term survival rate are poor due to underlying ischemic cardiomyopathy [25]. Pseudoaneurysm has also been observed after mitral valve surgery. Schuetz et al. treated nine patients with atrioventricular disruption after mitral valve procedures, showing that epicardial tissue sealing results in successful termination of bleeding and considerably improved survival compared with the standard surgical procedure [26].

The sutureless procedure is an attractive and simple treatment strategy for ischemic left ventricular rupture, but surgeons should be aware that it has a potential risk of pseudoaneurysm formation after surgery. Large fibrin glue sheets covering the entire infarcted myocardium could reduce the wall stress of the left ventricular lesion. Avoiding cardiopulmonary bypass might also contribute to successful results. Management after repair, including intra-aortic balloon pumping which decreases the afterload of the left ventricle, and other mechanical supports help to reduce the preload of the left ventricle, hopefully leading to better clinical results [27]. Additional serial echocardiographic studies are needed to diagnose complications so that patients can receive proper treatments.

## Conclusions

The sutureless technique can be a promising strategy for the treatment of ischemic rupture, but serial echocardiographic studies should be mandatory for diagnosing a left ventricular pseudoaneurysm formation thereafter.

## Abbreviations

Ant.: anterior wall of the left ventricle
ATCS: Ann Thrac Cardiovasc Surg
ATS: Ann Thorac Surg
CPB: cardio-pulmonary bypass
GTCS: Gen Thorac Cardiovasc Surg
IABP: perioperative intra-aortic balloon pumping
N.A.: not available
ICTS: Interact Cardiovasc Thorac Surg
Inf: inferior wall of the left ventricle
JCS: J Card Surg
JJTCS: Jpn J Thorac Cardiovasc Surg
JTCS: J Thorac Cardiovasc Surg
KG: Kyobu Geka
Lat.: lateral wall of the left ventricle
PA formation: Left ventricular pseudoaneurysm formation
PCPS: percutaneous cardio-pulmonary support
Per. Drain.: pericardial drainage
Post.: posterior wall of the left ventricle
Antero-lat: antero-lateral wall of the left ventricle
reop: reoperation
